# Analysis of the efficacy of an experimental expert system of medical prescription in reducing medical errors and excessive physician workload: a cross-sectional study

**DOI:** 10.1186/cc12633

**Published:** 2013-06-19

**Authors:** HH Shieh, ER Barreira, EJ Troster, SC Brassica, AC Ventura, PF Góes, I de COF Fernandes, DC de Souza, JC Fernandes, F Pereira das Chagas, R de Jesus, LO Zagne, FR Caino, AE Gilio, G Galvão de França, M Luglio, A Bousso

**Affiliations:** 1 University Hospital of Universidade de São Paulo, Hospital Cruz Azul de São Paulo, Instituto da Criança do HC-FMUSP, Hospital Israelita Albert Einstein, Vila Iara, São Paulo, SP, Brazil

## Introduction

Deaths attributable to preventable medical errors (PME) in hospitals exceed those caused by well-known life-threatening conditions, such as motor vehicle accidents, breast cancer, and AIDS [1]. The Institute of Medicine estimates that as many as 98,000 deaths are caused by PME every year [1]. The risks derived from PME are even more severe when they affect critically ill patients, or include medications that must be adjusted for the patient's body weight. Fatigue and work overload can represent a threaten to the patients' safety in pediatric ICUs [2]. Expert Systems (ES) [3], a branch of artificial intelligence, can be used to solve the problems related to medical prescription errors (MPE). Studies analyzing the role of ES in MPE are still lacking. The objective of this study was to compare the accuracy of an experimental ES with the written medical prescription.

## Methods

After signing an informed consent, pediatricians working in a university hospital were asked to write a medical prescription containing 10 different medications (maintenance fluids, adenosine, epinephrine, atropine, phenytoine, vancomycin, ceftazidime, amphothericin B, dobutamine, and fentanyl) for a hypothetical patient. The written medical prescription was compared with the ES prescription made by the same physicians, after a 2-minute training period. Statistical analysis was done using the χ^2^, Fisher's exact test, paired *t *test or Wilcoxon test (paired samples), whenever applicable. A significance level of 0.05 was used for all analyses.

## Results

Thirteen pediatric residents and seven attending physicians participated on the study; the mean time since medical graduation was 10.1 ± 9 years. Fifty-seven prescribing errors were detected on medical prescription (nine unreadable items, 23 omissions, six dosing errors, 14 dilution errors and five velocity of infusion errors) in comparison with one error of duplication of medication in the ES prescription (*P *<0.001).

## Conclusion

The medical prescription of critically ill pediatric patients deserves special attention. The use of an experimental ES required a short training period and resulted in a significant decrease in prescribing errors and physicians' workload. Nevertheless, this computerized approach is not error free, and double-checking must be performed by the prescriber physician.
